# An fMRI Study Using a Combined Task of Interval Discrimination and Oddball Could Reveal Common Brain Circuits of Cognitive Change

**DOI:** 10.3389/fpsyt.2021.786113

**Published:** 2021-12-20

**Authors:** María Sol Garcés, Irene Alústiza, Anton Albajes-Eizagirre, Javier Goena, Patricio Molero, Joaquim Radua, Felipe Ortuño

**Affiliations:** ^1^Department of Psychiatry and Clinical Psychology, Clínica Universidad de Navarra, Pamplona, Spain; ^2^Colegio de Ciencias Sociales y Humanidades, Universidad San Francisco de Quito USFQ, Quito, Ecuador; ^3^Instituto de Neurociencias, Universidad San Francisco de Quito USFQ, Quito, Ecuador; ^4^Instituto de Investigación Sanitaria de Navarra (IDISNA), Pamplona, Spain; ^5^Imaging of Mood and Anxiety Related Disorders (IMARD) Group, d'Investigacions Biomèdiques August Pi i Sunyer (IDIBAPS), CIBERSAM ES, Barcelona, Spain; ^6^Early Psychosis: Interventions and Clinical-Detection (EPIC) Lab, Department of Psychosis Studies, Institute of Psychiatry, Psychology and Neuroscience, King's College London, London, United Kingdom; ^7^Department of Clinical Neuroscience, Centre for Psychiatric Research and Education, Karolinska Institutet SE, Solna, Sweden

**Keywords:** timing, oddball, saliency network, cognitive control, fMRI, SDM-PSI meta-analysis

## Abstract

Recent functional neuroimaging studies suggest that the brain networks responsible for time processing are involved during other cognitive processes, leading to a hypothesis that time-related processing is needed to perform a range of tasks across various cognitive functions. To examine this hypothesis, we analyze whether, in healthy subjects, the brain structures activated or deactivated during performance of timing and oddball-detection type tasks coincide. To this end, we conducted two independent signed differential mapping (SDM) meta-analyses of functional magnetic resonance imaging (fMRI) studies assessing the cerebral generators of the responses elicited by tasks based on timing and oddball-detection paradigms. Finally, we undertook a multimodal meta-analysis to detect brain regions common to the findings of the two previous meta-analyses. We found that healthy subjects showed significant activation in cortical areas related to timing and salience networks. The patterns of activation and deactivation corresponding to each task type partially coincided. We hypothesize that there exists a *time and change-detection* network that serves as a common underlying resource used in a broad range of cognitive processes.

## Introduction

The notion that the ability of the human mind to perceive changes in the environment depends on perception of time can be traced right back to the ancient philosopher Aristotle (1). We now distinguish two aspects to the perception of time, and, in modern terminology, the word *timing* refers to two subjective experiences: the impression of passage of time and the impression of how much time has passed, for example, the perceived duration of an event (2). As temporality is related to change, timing might be expected to be related to saliency processing, that is, to the detection of change, for example, detection of a stimulus that deviates from the norm (3). It is well-documented that in humans, both timing and saliency processing are gradually acquired during normal development (4–6). Impairment in either ability has been associated with psychiatric and neurologic disease, such as schizophrenia (SZ) (3, 7–9). Other than the above correlations between timing and saliency processing, there are also a few studies that suggest the connection in a more direct manner (10–12). Both functions share neuroanatomical bases, and the way cognitive resources are allocated to each is joined (13).

The specific set of brain regions that constitute the neural substrate for timing have been elucidated by means of two meta-analysis studies of neuroimaging research (14, 15) and other relevant studies (16, 17). This infrastructure comprises cortical and subcortical regions: the supplementary motor area (SMA), the insula, the left inferior frontal region, the middle frontal gyrus, the left inferior parietal region, the left superior temporal gyrus, the right thalamus, the cerebellum, and the left putamen.

Timing clearly affects other cognitive functions, and this is reflected in the well-known Scalar Expectancy Theory (SET), for example, where timing is central to the model, which comprises a clock, working memory, and executive function (18, 19). It has been established that the cognitive domains for attention, working memory and executive functions require participation of functional aspects of timing and neuro-anatomical components of timing regions (20–22). A meta-analysis of functional neuroimaging studies (23) indicated that timing and other cognitive processes demanding cognitive control become interlinked when there is an increase in the level of difficulty or effort required.

For saliency processing, brain regions found to participate are the temporal parietal junction (TPJ), the anterior insula, the anterior middle frontal gyrus, the bilateral anterior cingulate cortex (ACC), and the SMA. The brain deals with saliency at two levels: there is a primitive level which operates before attention is invoked and at which change is simply detected, and there is a higher-order level which involves attention and links change with its implications such as goal-oriented responses (3). One of the main experimental designs used to examine the mechanisms of salience or novelty detection is the oddball paradigm (24, 25). This technique consists in repeating an auditory or visual stimulus, referred to as the *standard* stimulus, and occasionally including a different stimulus, known as the *deviant* or *oddball* stimulus. Electroencephalographically, there are two distinct event-related potentials (ERPs) observed during saliency detection. One of these is the so-called P300 wave, which is generated infrequently in response to a stimulus that has cognitive relevance (i.e., a target stimulus); the other ERP is the mismatch negativity (MMN) potential, which is generated when something changes (an oddball stimulus) in a repeated sequence of pre-attentional stimuli (26).

Oddball detection tasks can be used to assess a person's performance at change detection. Might timing tasks be used for the same end? Underlying this question or hypothesis is the idea, introduced in the first paragraph, that temporality is related to change, and so timing might be expected to be related to saliency processing. To try to answer the question, we might determine whether the Salience Network (SN) overlaps neuro-anatomically with the timing network, that is, attempt to identify the functional regions active during salience processing and timing in isolation of each other. Furthermore, it is also of interest to analyze the interrelationship between these two theoretically-separate cognitive functions: Are there any brain regions involved simultaneously by both?

We hypothesize that change detection paradigms such as oddball, context change deviants, and salience paradigms are actually alluding to a basic underlying function of time processing, specifically, time discrimination. To test this hypothesis, we sought to determine whether the neural networks activated in oddball studies and those activated in time discrimination studies are the same. The main goal of this study was to identify any structures activated during both timing and oddball tasks. To this end, we carried out two Seed-based d-mapping (SDM-PSI) meta-analyses of neuroimaging studies assessing the brain response to temporal discrimination and oddball tasks. After this, we undertook a multimodal meta-analysis to identify any possible common features in the findings of the two previous task-specific SDM-PSI meta-analyses.

## Materials and Methods

### Study Selection

Two independent systematic, comprehensive literature searches were conducted for functional magnetic resonance imaging (fMRI) studies in healthy volunteers using temporal discrimination and oddball tasks. The literature searches were conducted by means of the PubMed search engine from publications up to July 2021. A MeSH terms search strategy was adopted and filters for age and publication type were applied.

The search string for the timing studies search was: ((“Magnetic Resonance Imaging”[Mesh]) AND “Time Perception/physiology^*^”[Mesh] NOT “Mental Disorders/diagnosis”[Mesh]) NOT “Neurologic Manifestations/5diagnosis”[Mesh].

Two 132 papers were identified through a database search. Additionally, references form previous meta-analyses (14, 15, 27, 28) about time perception were reviewed for inclusion.

In the second search, for oddball studies, the search string (with corresponding keywords) used was: ((“Magnetic Resonance Imaging”[Mesh]) AND (“Event-Related”) AND ((“oddball”) OR (“target detection”)) NOT “Mental Disorders/diagnosis”[Mesh]) NOT “Neurologic Manifestations/diagnosis”[Mesh].

One 186 papers with oddball tasks were identified through database search and reviewed.

The studies that met the following inclusion criteria had been included in the analyses:

(a) studies using functional magnetic resonance imaging (fMRI) (any other neuroimaging technique were excluded, i.e., PET, SPECT); (b) studies using samples of healthy volunteers (studies that included both healthy and non-healthy subjects were excluded); (c) studies including more than five participants; (d) for the temporal domain, studies including a temporal discrimination perceptual supra-second task with at least one contrast of timing task (not contrast-rest); (e) for the saliency domain, studies including oddball task with standard vs. target contrast (not standard-novel or target novel); (f) studies performed a whole-brain analyses (i.e., articles that performed only region of interest (ROI) or small volume correction (SVM) analysis have been excluded); (g) studies that provide peak coordinates and statistical parametric maps in the publication; (h) studies that use a constant statistical threshold for all regions of the brain; (i) studies that are peer-reviewed articles reporting novel data on temporal or saliency processing (qualitative studies, case reports, reviews, or meta-analyses, were excluded).

### Systematic Review

We applied the PRISMA (Preferred Reporting Items for Systematic reviews and Meta-Analyses) guidelines (29) for the literature screening and final selection. The procedure flow diagrams are available within the files: “Supplementary Material 1.1” (for temporal discrimination and oddball). Two independent researchers conducted the PubMed searches. Initially, the title and abstract of the studies were screened for keywords, if the study was eligible, the full text was analyzed. When decisions about inclusion or exclusion criteria differed between reviewers', the final decision was resolved by a consensus between the two reviewers.

### The Meta-Analyses

The current study followed the most recent guidelines for the meta-analysis (30). We conducted two independent SDM-PSI meta-analyses of fMRI studies that assessed brain response during temporal discrimination and oddball tasks in healthy subjects. For the statistical process of meta-analysis, we used version 6.21 of Seed-based d Mapping software (SDM-PSI; Voxel-based meta-analysis *via* permutation of subject images (PSI): Theory and implementation for SDM, http://www.sdmproject.com) (31–34).

SDM-PSI is a statistical technique for meta-analysis of neuroimaging studies (fMRI, PET, VBM, or DTI) concerned with brain activity or structure. SDM-PSI improves on older meta-analysis methods [ALE or Kendel Density Analysis (KDA)] by including statistical approaches to deal with between-study heterogeneity, missing information (multiple imputation techniques), and *p*-value correction (standard permutation tests); in addition, SDM-PSI makes it possible to do meta-regression and multimodal meta-analysis (23, 34).

Based on the MetaNSUE algorithm, SDM-PSI first imputes datasets from each study's peak coordinates and statistical maps, which we obtained from the published papers (35). Next, each imputed dataset is meta-analyzed using standard random-effects models. The results for the multiple imputed datasets are then combined (using Rubin's rules). Finally, SDM-PSI applies standard permutations testing to obtain values of statistical significance. In this study, the thresholds applied to the results were the default values proposed by the software (*p* < 0.005 for the uncorrected *p*-values and *p* < 0.05 for Threshold Free Cluster Enhancement (TFCE) corrected *p*-values of main analyses).

### Multimodal Meta-Analysis for Timing and Oddball Studies

The results of the above two independent timing and oddball detection meta-analyses were subjected to multimodal meta-analysis with a view to identifying any regions that became activated or deactivated during both tasks. In order to achieve this, the two BOLD maps, corresponding to the response to timing and the response to oddball detection, were effectively laid on top of each other and compared. The software, however, does not generate a map simply by calculating the most probable overlap; it takes into account the estimates of error in *p*-values in each separate meta-analysis (36).

## Results

### Studies Included and Their Characteristics

Of the several 100 studies returned by PubMed, only 29 met the inclusion criteria. Of these, 11 examined timing (a total of 174 healthy subjects), and the other 18 studied oddball detection (225 healthy subjects), as shown in Tables 1, 2. From the oddball studies three studies were discarded due to some concerns in the reported coordinates and the lack of response from the authors after having contacted them for clarification. Therefore, only 26 studies were included (11 for timing and 15 for oddball). Please see Supplementary Material 1.2 for a report of the excluded studies and associated reasons for exclusion.

**Table 1:** Studies of timing in HC included in our SDM-PSI meta-analysis.

**References**	**Sample**	**Task**	**Included contrast**
Coull et al. (37)	12 HC	A visual time attention task (temporal discrimination)	Increase in attention to time and increase attention to color
Coull et al. (38)	14 HC	A visual temporal discrimination task	Time vs. color and color vs. time
Coull et al. (39)	16 HC	A visual temporal discrimination	Time vs. color
Lewis and Miall (40) Exp. B	8 HC	Temporal discrimination task	Time vs. length
Livesey et al. (41) Exp. A	10 HC	A visual temporal discrimination task	Time vs. color
Morillon et al. (42)	17 HC	A visual temporal estimation task	Duration vs. color and color vs. duration
Pfeuty et al. (43)	29 HC	A visual temporal estimation task	Duration vs. color and color vs. duration
Pouthas et al. (44)	6 HC	A visual temporal estimation task	Long vs. short duration
Rao et al. (45)	17 HC	An auditory temporal discrimination task	Time vs. control
Smith et al. (46)	20 HC	A visual temporal discrimination task	Temporal vs. order and order vs. discrimination
Wiener et al. (47)	25 HC	A visual temporal discrimination task	Time vs. color and color vs. time

*HC, healthy controls*.

**Table 2:** Studies of oddball in HC included in our SDM-PSI meta-analysis.

**References**	**Sample**	**Task**	**Included contrast**
Eichele et al. (48)	15 HC	An auditory oddball task	Target vs. standard
Fajkus et al. (49)	34 HC	A visual 3-stimulus oddball task	Target (infrequent) vs. baseline (frequent)
Friedman et al. (50)	15 HC	Auditory oddball task	Target vs. baseline
Gur et al. (51)	36 HC	A visual 3-stimulus oddball task	Target vs. standard
Horovitz et al. (52)	7 HC	An auditory oddball task	Target vs. baseline
Huettel et al. (53)	14 HC	A visual oddball task	Target vs. frequent
Linden et al. (54)	5 HC	An auditory oddball task	Target vs. non-target
Mantini et al. (55)	13 HC	A visual oddball task	Rare vs. frequent
Menon et al. (56)	11 HC	An auditory oddball task	Target vs. frequent
Mulert et al. (57)	9 HC	An auditory oddball task	Target vs. non-target
Müller et al. (58)	16 HC	An auditory oddball task	Target vs. standard
Petit et al. (59)	8 HC	An auditory oddball task	Attended deviant tones compared to standard tones
Sabri et al. (60)	17 HC	An auditory oddball task	Main effect of deviance vs. standards
Stevens et al. (61)	10 HC	An auditory oddball task	Standard and target
Warbrick et al. (62)	15 HC	A visual hybrid two choice reaction time–oddball task	Target vs. standard

*HC, healthy controls*.

### Meta-Analysis Results for Timing Studies

**Figure 1:**
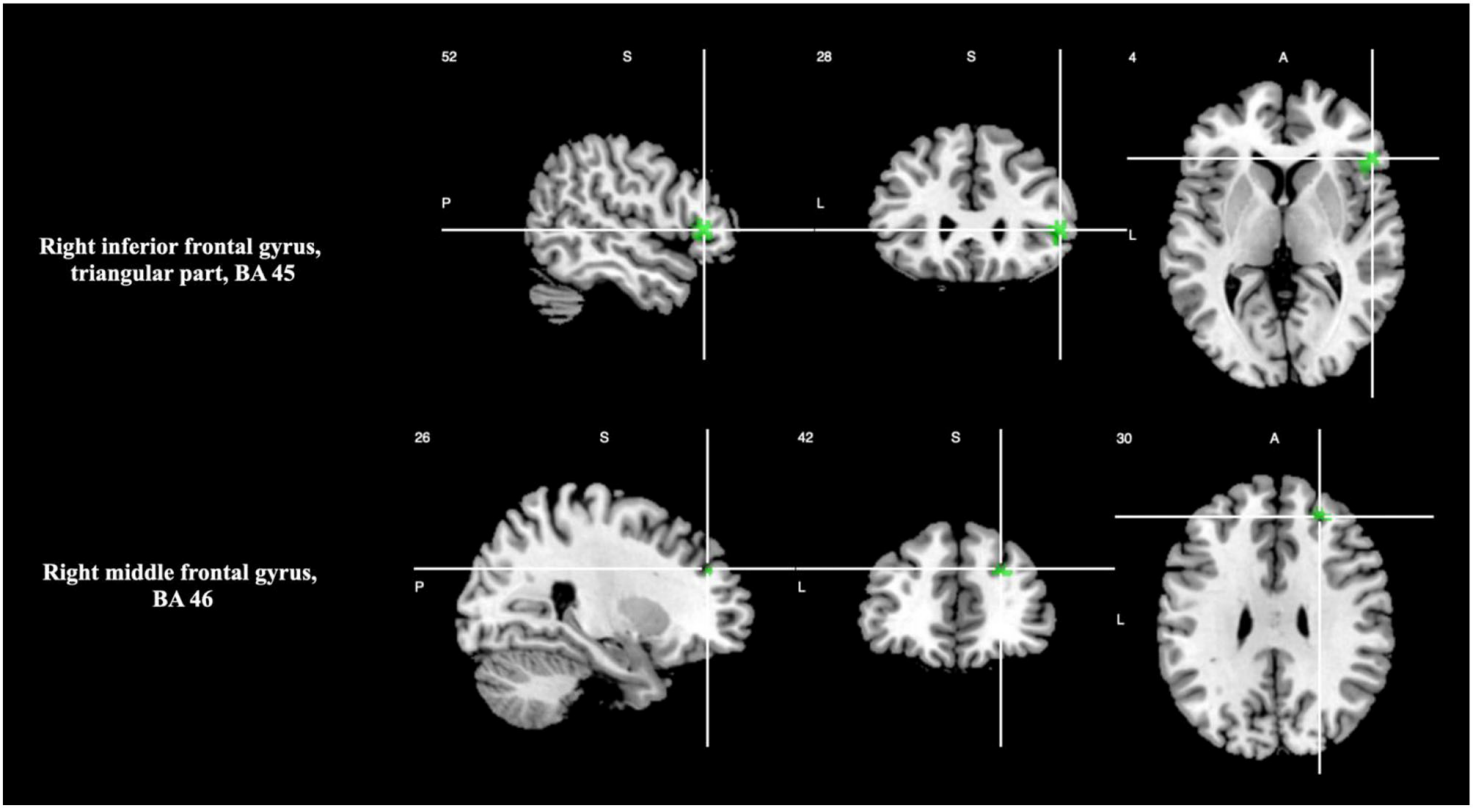
Brain regions engaged during temporal discrimination tasks.

There was significant activation in the right inferior frontal gyrus, triangular part (BA 45), and right middle frontal gyrus (BA 46), as shown in Table 3 and Figure 1. Please see Supplementary Material 2.1 for breakdown analysis. No hypoactivations were found. In a supplementary analysis applying *p* < 0.025 for the uncorrected *p*-values of the main analyses and *p* < 0.05 TFCE after the FWE correction, Supplementary Motor Area (SMA) (BA 6) activation was shown.

**Table 3:** Brain regions engaged during timing tasks.

**Location**	**Peak**			
	**MNI**	***Z***	***P***	**Voxels**
Right inferior frontal gyrus, triangular part, BA 45	52, 28, 4	5.545	0.000999987	1,487
Right middle frontal gyrus, BA 46	26, 42, 30	4.668	0.000999987	678

*Threshold: voxel P < 0.0500, peak SDM-Z > 1.000, cluster extent size ≥10 voxels. Breakdown regions with <10 voxels are not reported*.

### Meta-Analysis Results for Oddball Studies

**Figure 2:**
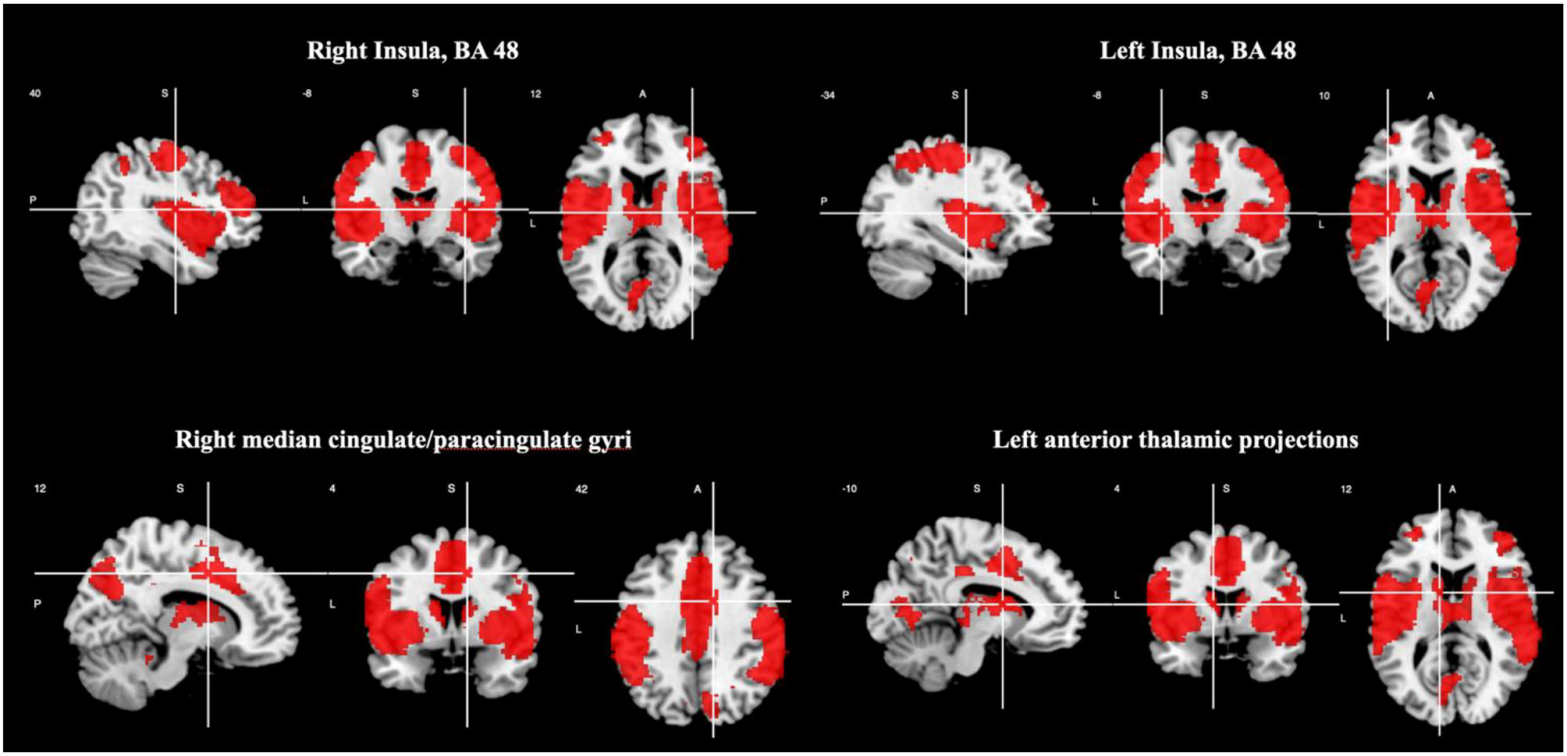
Brain regions engaged during oddball tasks.

There was increased activation in the right and left insula (BA 48), right median cingulate/ paracingulate gyri, and left anterior thalamic projections as shown in Table 4 and Figure 2. Please see Supplementary Material 2.2 for breakdown analysis. No hypoactivations were found.

**Table 4:** Brain regions engaged during oddball detection tasks.

**Location**	**Peak**			
	**MNI**	***Z***	***P***	**Voxels**
Right insula, BA 48	40, −8, 12	6.095	0.000999987	19,652
Left insula, BA 48	−34, −8, 10	8.248	~0	13,842
Right median cingulate/ paracingulate gyri	12, 4, 42	6.779	0.000999987	5,347
Left anterior thalamic projections	−10, 4, 12	4.619	0.007000029	1,193

*Threshold: voxel P < 0.0500, peak SDM-Z > 1.000, cluster extent size ≥10 voxels. Breakdown regions with <10 voxels are not reported*.

### Multimodal Meta-Analysis Results for Combined Timing and Oddball Studies

**Figure 3:**
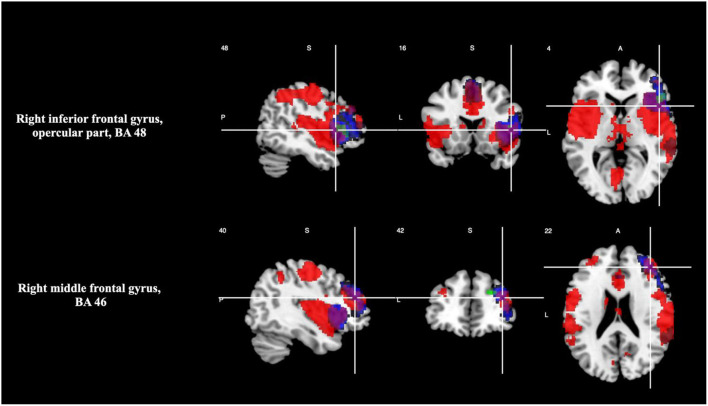
Overlap between brain regions engaged during timing and oddball tasks (magenta). Brain regions in neurological convention showing areas with statistically signification activation only during timing tasks (green) and during oddball tasks (red).

At TFCE corrected *p* < 0.05 level of statistical significance, areas with overlapping of activation were right inferior frontal gyrus, opercular part (BA 48), and right middle frontal gyrus (BA 46), as shown in Table 5 and Figure 3. Please see Supplementary Material 2.3 for breakdown analysis. No hypoactivations were found.

**Table 5:** Brain regions engaged during timing and oddball detection tasks.

**Location**	**Peak**	
	**MNI**	**Voxels**
Right inferior frontal gyrus, opercular part, BA 48	48, 16, 4	1,012
Right middle frontal gyrus, BA 46	40, 42, 22	271

*Threshold: voxel P < 0.0500, peak SDM-Z > 1.000, cluster extent size ≥10 voxels. Breakdown regions with <10 voxels are not reported*.

## Discussion

The main finding of the current study, that there was common engagement of certain neural regions when healthy subjects were doing timing and oddball tasks, provides evidence that timing and salience processing are connected, as long as a cognitive effort is involved.

Our interest in the idea that timing is connected with detection of change originated with the observation that the timing activation-deactivation pattern changes with the level of cognitive effort required by the task, which led on to the suggestion that there might be a temporal-cognitive control network (63). As salience processing is characterized by change detection, we hypothesized that timing regions overlap with salience processing regions.

Timing is a function implicated in multiple and diverse cognitive, affective, and regulatory processes. Previous studies have shown that the relationship between time perception and a range of cognitive functions is mediated by the increase of cognitive effort demanded by the task (64). Performing any cognitive task requires constant modulation of the level of effort to cope with changes occurring around us (65). In previous studies (9, 63, 66), we looked into the recruitment of temporal circuits in a wide range of cognitive processes involving cognitive control in healthy subjects finding a functional link and overlap between regions whenever a change in the level of cognitive effort occurred. Thus, it seems that appropriate cognitive functioning across different levels of difficulty requires the participation of functional and anatomical components of time perception (64). These previous findings of our team are consistent with the study of Livesey et al. (41), who reported that an increase in the difficulty of non-temporal tasks invoked the participation of the timing network. This reinforces the point that timing and change detection are interrelated. Thus, any mental process that involves change detection or that is activated by detection of change needs to use timing processing, which, we hypothesize, is provided by an underlying temporal-salience network.

In a previous SDM meta-analysis study, we addressed the question of whether a dysfunctional timing/change detection network underlies the cognitive impairment observed in SZ. We found a partial coincidence of dysfunction (hypoactivation in cortical and subcortical areas) during timing and change-detection tasks in SZ compared to healthy subjects (9). The study also suggested that there existed a group of brain regions that engaged both during timing and oddball tasks in normal cognition, and it was in order to investigate this further, that we performed the current study. More precisely, the objective of the current meta-analysis is to examine whether traditional taxonomies of timing (perceptual and supra-second) and salience functions purporting discrete modular cognitive domains are supported by a superordinate cognitive control system that is engaged during the performance of a range of timing and oddball tasks.

For timing tasks, our results, based on a larger number of published fMRI data sets than our previous meta-analysis (15), which was also carried out to explore the neuroanatomical basis of timing, further confirm the participation of regions of the temporality circuit. These regions include the right middle (BA 46) and inferior frontal gyrus, triangular part (BA 45).

In contrast to our previous meta-analysis, the current one did not detect the cerebellum, parietal, temporal, and subcortical regions. An interpretation for this failure is that unlike the first, the present meta-analysis included only studies with perceptual and suprasecond temporal discrimination tasks (requires participants to compare two-time intervals). Our findings are also in agreement with those shown by Wiener et al. (14), Nani et al. (27), and Cona et al. (28). For example, the mentioned frontal (BA45, BA46) is involved in their analysis of supra-second perceptual timing tasks. In contrast the absent regions in our analysis (cerebellum, parietal, temporal, and subcortical) are showed in their analysis of sub-second or motor timing data dimensions: stimulus duration (sub- vs. supra-second) and the nature of response (motor vs. perceptual). Therefore, differences regarding the participation of regions congruent with the temporality circuit between our findings and those reported by previous meta-analyses may be due to the inclusion of studies that address different modalities of timing tasks.

Our analysis of temporal discrimination tasks yielded no significant activation of the SMA at the level of *p* < 0.005 uncorrected *p*-values and TFCE *p* < 0.05 corrected *p*-values, failing to replicate the results of numerous meta-analyses (14, 27, 28). However, SMA activation was shown at the level of *p* < 0.025 uncorrected *p*-values and TFCE *p* < 0.05 corrected *p*-values. A possible explanation for this discrepancy could be found in the test of spatial convergence used to estimate the statistical significance of the results by most of the available CBMA methodologies. As recently shown, this test of spatial convergence may rely on assumptions commonly not meet by real data. The violation of such assumptions has been proven to make the results of this permutation test either conservative or liberal, depending on which assumption was not met (34). Moreover, the statistical significance of the results estimated by the test has been proven to be sensitive to the number of effects present in the brain: the more effects present, the lower the significance estimated (34). We apply a new algorithm (SDM-PSI) that includes standard permutations of subject images, accurate control of the few, and the usage of Threshold-free Cluster Enhancement (TFCE) (67). Thus, SDM-PSI overcomes the drawbacks by the standard permutations test in order to estimate the statistical significance *p*-values, increasing sensibility, and power.

There was a pattern of activation in several superior, middle and lower frontal regions, in regions of the parietal and occipital lobes and insula with oddball tasks. These areas correspond with the areas that mediate the salience circuit (such as the anterior cingulate cortex, anterior insula, and sub-lenticular extended amygdala) (68).

The meta-analyses for just timing tasks and for just oddball tasks, indicate that oddball style tasks engage some regions associated with timing. The multimodal meta-analysis for combined timing and oddball studies confirms this observation: subjects showed significant (*p* < 0.05) activation in timing-related areas (particularly, the middle and inferior frontal gyrus) and at the local peak level of the insula. Not all neural regions of both the timing and salience processing circuits were detected as being activated; however, the degree of overlapping of activation patterns recorded during timing and oddball tasks supports the hypothesis that an integral timing and salience/change processing circuit exists.

The existence of an integral time and change processing circuit leads to the idea that such a circuit can be used by and underlies other kinds of cognitive task: any task involving change detection. Because cognitive control depends on the detection of change in the level of cognitive effort demanded, we suggest that cognitive control, too, must invoke the time and change processing circuit, and this idea leads on further to a putative temporal-salience-cognitive control network.

Our findings show region similarities with three of the six differentiated neural circuits suggested by Williams (68) (“Default Mode,” “Salience,” “Threat,” “Reward,” “Attention,” and “Cognitive Control”), specifically with Salience, Attention and Cognitive Control networks. An implication of this is that tasks that make use of the Salience, Attention, and Cognitive Control networks involve aspects of temporality and/or change.

The observation that the processing of timing and the processing of change detection uses the same set of brain regions can be explained by supposing that both types of processing depend on the same or similar set of cognitive abilities; both require, for example, working memory and attention, and both involve executive functions. Our results, to some degree, support such an explanation. We found that brain regions (such as frontal) that are classically thought of as being cognitive-related were active during timing tasks, and regions (such as frontal regions and insula) that are regarded as being principally concerned with timing were busy during oddball tasks. Both groups of regions are partially subsumed within the circuit for attention, which includes regions in the medial superior frontal cortices, anterior insula, anterior inferior parietal lobule and precuneus (68).

Matthews and Meck (69) proposed a framework that connects time perception with other cognitive domains by suggesting a processing principle that outlines some of the links between them. Even though they claim that non-temporal variables affect subjective estimation of time, they do not rule out the possibility that subjective estimation of duration may affect perceptual saliency of stimuli and information extraction, as is implied to be the case under our hypothesis that the time and change circuit modulates other non-temporal cognitive functions.

Karmakar and Buenomano (70) examine an alternative to a clock-based timing mechanism for mental processing. They propose that the neural circuits responsible for timing, as well as intrinsically representing time, primarily process other non-temporal information such as stimulus features. Regarding this view, Muller and Nobre (71) suggest that a dedicated timing mechanism may be embedded in the processing of other stimuli attributes; therefore, timing processes take place in time, without necessarily coding time *per se*. Gell (72) postulates that perception is intrinsically time-perception, as time is an inextricable feature of any perceptual process. Previously, Fraisse (73) proposed that time perception resides in the detection of change through the integration of stimuli.

### Salience Network

Regarding the relationship between the salience network and cognitive effort, Lamichhane et al. (74) showed that SN activity is linked to task difficulty, as sensory integration of a salient stimulus to the task requires a higher level of effort and network engagement. Our results concur. Lamichhane et al. (74) found evidence of a relationship between an increase in activity in the anterior insula (AI) along with in the dorsal anterior cingulate cortex (dACC) and an increase in task difficulty, suggesting a central role of the AI in the integration of sensory inputs and cognition. The information processing described not only depends on the AI alone; it is supported by the AI's frontal, parietal, and temporal efferent and afferent projections and by its functional connectivity with other networks (74–76).

The human insula is implicated in multiple brain functions (77). Nieuwenhuys (78) described around 20 different insular functions. The AI is a structural member of the SN, responsible for target detection (79). Within saliency functions, the AI is involved in bottom-up novelty detection, modulation of autonomic reactivity to salient stimuli, and accessibility to attentional working memory resources during saliency detection (79). Additionally, the AI coordinates activity changes across networks, an aspect of cognitive control to which the SN contributes (80).

Note that our results found activation of the left insula without specification of subdivisions; the left insula includes both anterior and posterior components (81).

### Cognitive Control

The activation pattern for cognitive control overlaps and extends beyond regions that are expected for a “multiple-demand system” operative for attentional activity (82). This suggests there is some other common process that is even more widely demanded than attention.

Several studies (64, 83, 84) suggest the existence of a superordinate cognitive control network that involves DLPFC, medial frontal cortex (including the anterior cingulate cortex [ACC]), parietal cortex, motor areas, and cerebellum) that support a broad range of high-level cognitive functions (that is, the executive functions). Our team's previous studies provided evidence of the existence of the aforementioned network and indicated it included additional regions [medial frontal (SMA), temporal insula, and basal ganglia]. We proposed that the network was essentially a temporal-cognitive control circuit rather than a circuit specifically controlling executive functions (63, 64). This idea of a temporal-cognitive control network derived from the observations that there was participation of various cognitive processes during time perception tasks and that there was an engagement of temporal processing during non-temporal cognitive tasks when those tasks switched in level of difficulty (64).

Some researchers regard the various areas that comprise the multiple demand network (frontal and parietal) as part of a single dedicated network. There is, however, evidence that these regions also participate in partially separate control networks: the cingulo-opercular (CO), frontoparietal (FP) (85), salience (86), and dorsal and ventral attention networks (87). On the basis of findings in transcranial magnetic stimulation studies (88), it is reasonable to suggest that these networks dynamically interact and integrate into certain contexts when task complexity increases. This interaction may be required for the coordination, updating, and maintenance of information relevant to the task as well as for execution of the task (89). Of the above-mentioned networks, the SN may be involved in regulating changes in functional connectivity between other networks throughout the brain (79). Gratton et al. (90) discuss how network interactions occur and whether some particular regions are critical for this interaction; the authors propose that there are specific hubs that act as mediators of interaction and that play a role in information transfer between networks.

We propose that the timing network implied by our results be regarded as one more of the partially separate control networks discussed above. Under this view, and with respect to executive cognitive functions, the timing network interacts with other brain networks to deal with changes in task difficulty. In our results, not all of the structures of the overarching cognitive control network were activated. So we suggest that those structures that were activated and that overlapped with structures in the saliency network be provisionally regarded as the hubs of the timing network because they are the structures that appear to participate in the timing network's different interactions with other regions.

### Implications for Schizophrenia

Schizophrenia is associated with deficits in multiple cognitive processes. Previous meta-analyses have shown an interrelationship between temporal, cognitive control (63) and saliency processing in SZ (9). From these findings, we proposed the existence of a temporal-cognitive control network and a temporal-change detection network. Furthermore, exploring if a relationship between these networks is also present in normality allowed us to strengthen the hypothesis that timing is a primary cognitive domain that underlies and influences other cognitive processes. Moreover, timing structures overlap and contribute to other networks, and proper interaction is necessary for normal cognitive functioning. Therefore, assessing timing deficits as a potential biomarker for SZ may have a clinical implication in the pathology's diagnosis and treatment course.

### Additional Considerations

The study of timing is challenging due to the marked individual differences as well as to the liability across changes in experimental tasks (91). To address this heterogeneity in research and study, new integrative approaches are emerging. Researchers are increasingly supplementing the traditional approaches and simultaneously applying behavioral, neuroimaging, pharmacological, and genetic techniques. Individual differences could be linked to time perception through the study of genetic variation as human cognitive performance is highly variable and under strong genetic control, as well brain synchronism in time perception has shown genetic influence (92). Studies of different disorders related to cognitive impairment (Alzheimer's, schizophrenia, and muscular dystrophy) have shown that animal models are suitable for studying several dimensions of genetic, behavioral, and neural pathways underlying the expression of psychopathology (93–95). The integration of methods will allow a better understanding of neurotypical and psychopathological time processing.

In summary, our findings support the hypothesis that there exists a *time and change-detection network* that overlaps with other cognitive networks and is used during cognitive tasks in general for timing and detection of change and is also evoked whenever a variation of task difficulty occurs. Furthermore, we believe that this framework can provide an account of timing in neurotypical adults and may provide novel insights into the neural basis of disorders of timing, as a primary cognitive domain that underlies or broadly influences other cognitive processes.

### Limitations

Meta-analysis as a technique has various limitations. The data is less accurate for coming from various independent studies. Results tend to be less precise, especially with peak-based meta-analysis, because results are based on published coordinates as opposed to raw statistical brain maps. With a voxel-wise approach, as used in this study, the technique errs toward a failure to detect a region rather than to give false positives (34). There was only a small number of publications appropriate for inclusion in our meta-analysis, and therefore the results need to be regarded with circumspection until confirmed by further study.

Another important consideration when interpreting our results is that while fMRI techniques allow us to detect regions involved in cognitive functions from which we infer relationships, we cannot establish functional interconnectivity in a sense defined by graph theory.

The present study focuses on the relationship between temporal discrimination and attentional change detection, but it would also be interesting to study the relationship between temporal discrimination and the neurophysiological paradigms of evoked potentials such as MMN. Unfortunately, we were unable to include MMN studies in our meta-analysis because we found only three papers that complied with the inclusion criteria.

## Conclusions

By conducting two independent paradigm-specific meta-analyses and a multimodal conjunct analysis, the current study explores the relationship between the brain networks involved during oddball and time discrimination tasks in healthy subjects. We propose that timing circuits underlie any cognitive task as long as it involves change detection. Our results from this and previous studies suggest that timing is related to cognitive control and salience detection as both implicate change: in cognitive effort and in perceptual content, respectively. These findings support the hypothesis that a wider time and change-detection network that serves as a common underlying resource for other cognitive domains exists. However, our results are preliminary, and further studies are required to assess the specific role of timing in healthy and altered cognition. To verify our hypothesis and to enable application of graph theory, neuroimaging studies into concurrent execution of oddball and time discrimination tasks are required. Meanwhile, the hypothesis that there exists a time and change-detection network might be useful to initiatives focussing on improving our knowledge of the connectome in both health (i.e., functional Human Connectome) and disease [i.e., The Research Domain Criteria (RdoC)] (96).

Common models of human cognition have been proposed as candidates for the large-scale brain functional architecture. These models can be used for reproducing human-like artificial intelligence for research and clinical purposes (97). By identifying the cognitive primary domains that subserve the functionality of higher cognition, it is possible to refine neuroarchitecture models and establish a framework to further understand cognitive impairment in several psychopathologies. Unraveling the building blocks of cognition, as we proposed in the current study with the change-detection network, would contribute to expand our comprehension of cognitive neuroscience and find new approaches for clinical intervention.

## Data Availability Statement

The original contributions presented in the study are included in the article/Supplementary Material, further inquiries can be directed to the corresponding authors.

## Ethics Statement

Ethical review and approval was not required for the study on human participants in accordance with the local legislation and institutional requirements. Written informed consent for participation was not required for this study in accordance with the national legislation and the institutional requirements.

## Author Contributions

MG, IA, and FO designed and researched the review and meta-analysis. MG, AA-E, and JR conducted the two independent signed differential mapping (SDM) meta-analyses, developed the Images, and supervised the correct application of PRISMA criteria to select articles. MG wrote the first draft of the manuscript and re-drafted the manuscript. IA, FO, PM, and JG commented and corrected the manuscript and commented and approved the second draft. All authors contributed substantially to the manuscript, addressing different tasks, and have approved the final manuscript.

## Funding

This research has been financed by a project of the Carlos III Health Institute. Health Institutions (Project File Number: PI17/00240). Funded by Instituto de Salud Carlos III and co-funded by European Union (ERDF/ESF, “A way to make Europe”). Additional funding was provided by Universidad San Francisco de Quito, USFQ-COCISOH Grants.

## Conflict of Interest

The authors declare that the research was conducted in the absence of any commercial or financial relationships that could be construed as a potential conflict of interest.

## Publisher's Note

All claims expressed in this article are solely those of the authors and do not necessarily represent those of their affiliated organizations, or those of the publisher, the editors and the reviewers. Any product that may be evaluated in this article, or claim that may be made by its manufacturer, is not guaranteed or endorsed by the publisher.
