# Supplementary material for: An fMRI Study Using a Combined Task of Interval Discrimination and Oddball Could Reveal Common Brain Circuits of Cognitive Change
Source: Front Psychiatry. 2021 Dec 20;12:786113. doi: 10.3389/fpsyt.2021.786113 (PMC8721204; doi:10.3389/fpsyt.2021.786113)
Supplement: Supplementary file 1 [file Data_Sheet_1.docx]

Supplementary Material

# Supplementary Data

## PRISMA Flow Charts

**PRISMA 2020 flow diagram for timing studies**

**Identification of studies via databases**

Records removed *before screening*:

Duplicate records removed

(n = 59)

Records removed for other reasons (n = 50)

Records identified from:

PubMed Database (n = 232)

Other sources (previous meta-analyses) (n = 170)

**Identification**

Records screened

(n = 293)

Records excluded by human

(n = 171)

Reports not retrieved

(n = 100)

Reports sought for retrieval

(n = 122)

**Screening**

Reports excluded:

Wrong task (only temporal discrimination task included) (n =11)

Reports assessed for eligibility

(n = 22)

**Included**

Studies included in review

(n = 11)

**PRISMA 2020 flow diagram for oddball studies**

**Identification of studies via databases**

Records identified from:

Databases (n = 186)

Records removed *before screening*:

Duplicate records removed

(n = 0)

**Identification**

Records screened

(n = 186)

Records excluded by human

(n = 36)

Reports sought for retrieval

(n = 150)

Reports not retrieved

(n = 100)

**Screening**

Reports excluded:

Methodology (n = 4)

Wrong task (n = 7)

Wrong analysis (n = 8)

No coordinates (n = 5)

ROI (n = 3)

Wrong contrast (n = 5)

Incomplete data, no answer from authors after contact

(n = 3)

Reports assessed for eligibility

(n = 50)

Studies included in review

(n = 15)

**Included**

*From:*  Page MJ, McKenzie JE, Bossuyt PM, Boutron I, Hoffmann TC, Mulrow CD, et al. The PRISMA 2020 statement: an updated guideline for reporting systematic reviews. BMJ 2021;372:n71. doi: 10.1136/bmj.n71

For more information, visit: <http://www.prisma-statement.org/>

## Candidate Tables: Reports Assess for Eligibility

**Timing**

| No. | PMID | Authors | Study | Selection | Reason |
| --- | --- | --- | --- | --- | --- |
| 1 | 29662447 | Apaydın et al. (2018) | Neural mechanisms underlying time perception and reward anticipation. | No | Wrong task: Temporal prediction, not temporal discrimination |
| 2 | 27313526 | Carvalho et al. (2016) | Time-perception network and default mode network are associated with temporal prediction in a periodic motion task. | No | Wrong task: Temporal prediction, not temporal discrimination |
| 3 | 10689056 | Coull et al. (2000) | Orienting attention in time: Behavioural and neuroanatomical distinction between exogenous and endogenous shifts. | No | Wrong task: Temporal prediction, not temporal discrimination |
| 4 | 15001776 | Coull et al. (2004) | Functional anatomy of the attentional modulation of time estimation. | Yes |  |
| 5 | 18457512 | Coull et al. (2008) | Timing, storage, and comparison of stimulus duration engage discrete anatomical components of a perceptual timing network. | Yes |  |
| 6 | 23175824 | Coull et al. (2012) | Dopamine precursor depletion impairs timing in healthy volunteers by attenuating activity in putamen and supplementary motor area. | Yes |  |
| 7 | 22964490 | Coull et al. (2013) | Functional anatomy of timing differs for production versus prediction of time intervals. | No | Wrong task: Temporal prediction, not temporal discrimination |
| 8 | 26226079 | Coull et al. (2015) | SMA Selectively Codes the Active Accumulation of Temporal, Not Spatial, Magnitude. | No | Wrong task: Temporal prediction, not temporal discrimination |
| 9 | 27431757 | Coull et al. (2016) | Differential roles for parietal and frontal cortices in fixed versus evolving temporal expectations: Dissociating prior from posterior temporal probabilities with fMRI. | No | Wrong task: Temporal prediction, not temporal discrimination |
| 10 | 12387798 | Gandour et al. (2002) | Neural circuitry underlying perception of duration depends on language experience. | No | Wrong task: No control coordinates |
| 11 | 15464351 | Harrington et al. (2004) | Neural representation of interval encoding and decision making. | No | Wrong task: Mixed memory task |
| 12 | 19778958 | Harrington et al. (2010) | Neural modulation of temporal encoding, maintenance, and decision processes. | No | Wrong task: Mixed memory task |
| 13 | 12887983 | Lewis & Miall (2003) (Exp. B) | Brain activation patterns during measurement of sub- and supra-second intervals. | Yes |  |
| 14 | 16934301 | Livesey et al. (2007) Exp. A | Time perception: manipulation of task difficulty dissociates clock functions from other cognitive demands. | Yes |  |
| 15 | 14597297 | Lux et al. (2003) | Neural mechanisms associated with attention to temporal synchrony versus spatial orientation: An fMRI study. | No | Wrong task: Temporal prediction, not temporal discrimination |
| 16 | 19940175 | Morillon et al. (2009) | Three stages and four neural systems in time estimation. | Yes |  |
| 17 | 25366500 | Pfeuty et al. (2015) | fMRI identifies the right inferior frontal cortex as the brain region where time interval processing is altered by negative emotional arousal. | Yes |  |
| 18 | 15852471 | Pouthas et al. (2005) | Neural network involved in time perception: an fMRI study comparing long and short interval estimation. | Yes |  |
| 19 | 11224550 | Rao et al. (2001) | The evolution of brain activation during temporal processing. | Yes |  |
| 20 | 14527594 | Smith et al. (2003) | A right hemispheric frontocerebellar network for time discrimination of several hundreds of milliseconds. | Yes |  |
| 21 | 28286475 | Ustun et al. (2017) | Neural networks for time perception and working memory. | No | Wrong task: Temporal prediction, not temporal discrimination |
| 22 | 24269802 | Wiener et al. (2014) | Individual differences in the morphometry and activation of time perception networks are influenced by dopamine genotype | Yes |  |

**Oddball**

| No. | PMID | Authors | Study | Selection | Reason |
| --- | --- | --- | --- | --- | --- |
| 1 | 12421658 | Ardekani et al. (2002) | Functional magnetic resonance imaging of brain activity in the visual oddball task | No | No coordinates available |
| 2 | 19900561 | Arja SK et al. (2010) | Changes in fMRI magnitude data and phase data observed in block-design and event-related tasks | No | Wrong analysis: Magnitude, positive phase |
| 3 | 17295312 | Bénar et al. (2007) | Single-trial analysis of oddball event-related potentials in simultaneous EEG-fMRI | No | No coordinates available |
| 4 | 15496671 | Bledowski et al. (2004) | Localizing P300 generators in visual target and distractor processing: a combined event-related potential and functional magnetic resonance imaging study | No | Wrong analyses: Source Analysis Constrained |
| 5 | 11532888 | Braver et al. (2001) | Anterior cingulate cortex and response conflict: effects of frequency, inhibition and errors | No | Wrong analysis: Dysconjuction analyses |
| 6 | 30414982 | Cacciaglia et al. (2019) | Auditory predictions shape the neural responses to stimulus repetition and sensory change | No | Wrong task: MMN |
| 7 | 25556848 | Cacciaglia et al. (2015) | Involvement of the human midbrain and thalamus in auditory deviance detection | No | Wrong task: MMN |
| 8 | 16246587 | Calhoun et al. (2006) | Neuronal chronometry of target detection: fusion of hemodynamic and event-related potential data | No | Wrong analyses: ICA |
| 9 | 23313569 | Campanella et al. (2013) | BOLD response to deviant face detection informed by P300 event-related potential parameters: a simultaneous ERP-fMRI study | No | Wrong analyses: Null conjuction |
| 10 | 12482088 | Clark (2002) | Orthogonal polynomial regression for the detection of response variability in event-related fMRI | No | Wrong analysis: Orthogonal Polynomials analyses |
| 11 | 16768376 | Crottaz-Herbette & Menon (2006) | Where and when the anterior cingulate cortex modulates attentional response: combined fMRI and ERP evidence | No | ROI |
| 12 | 11305902 | Desjardins et al. (2001) | Removal of confounding effects of global signal in functional MRI analyses | No | Methodology: Global signal |
| 13 | 19015088 | Dichter et al. (2009) | Mapping social target detection with functional magnetic resonance imaging | No | Wrong contrast: Novel |
| 14 | 16314575 | Eichele et al. (2005) | Assessing the spatiotemporal evolution of neuronal activation with single-trial event-related potentials and functional MRI | Yes |  |
| 15 | 25989317 | Fajkus et al. (2015) | An fMRI investigation into the effect of preceding stimuli during visual oddball tasks | Yes |  |
| 16 | 10860792 | Fize et al. (2000) | Brain areas involved in rapid categorization of natural images: an event-related fMRI study | No | Wrong task: Visual go/no-go categorization task |
| 17 | 18465750 | Friedman et al. (2009) | The brain's orienting response: An event-related functional magnetic resonance imaging investigation | Yes |  |
| 18 | 17133387 | Gur et al. (2007) | Hemodynamic responses in neural circuitries for detection of visual target and novelty: An event-related fMRI study | Yes |  |
| 19 | 19492302 | Habermeyer et al. (2009) | Neural correlates of pre-attentive processing of pattern deviance in professional musicians | No | Wrong task: Preattentive |
| 20 | 12165350 | Horovitz et al. (2002) | Correlations and dissociations between BOLD signal and P300 amplitude in an auditory oddball task: a parametric approach to combining fMRI and ERP | Yes |  |
| 21 | 15053955 | Huettel et al. (2004) | Dynamic and strategic aspects of executive processing | Yes |  |
| 22 | 10547332 | Kang et al. (1999) | An event-related fMRI study of implicit phrase-level syntactic and semantic processing | No | Wrong task: N400 and P300 |
| 23 | 11321614 | Kiehl et al. (2001) | Neural sources involved in auditory target detection and novelty processing: an event-related fMRI study | No | Incomplete data, no answer from authors after contact |
| 24 | 15808990 | Kiehl et al. (2005) | An adaptive reflexive processing model of neurocognitive function: supporting evidence from a large scale (n = 100) fMRI study of an auditory oddball task | No | ROI |
| 25 | 11707088 | Kruggel et al. (2001) | Hemodynamic and electroencephalographic responses to illusory figures: recording of the evoked potentials during functional MRI | No | ROI |
| 26 | 18193453 | Laufer at al. (2008) | Sensory and cognitive mechanisms of change detection in the context of speech | No | Wrong task: MMN |
| 27 | 10601000 | Linden et al. (1999) | The functional neuroanatomy of target detection: an fMRI study of visual and auditory oddball tasks Exp A | Yes |  |
| 28 | 22377443 | Mangalathu-Arumana et al. (2012) | Within-subject joint independent component analysis of simultaneous fMRI/ERP in an auditory oddball paradigm | No | Wrong analysis: jilCA |
| 29 | 18793734 | Mantini et al. (2009) | Large-scale brain networks account for sustained and transient activity during target detection | Yes |  |
| 30 | 21620907 | Melcher et al. (2011) | How negative affect influences neural control processes underlying the resolution of cognitive interference: an event-related fMRI study | No | Wrong task: Mixed oddball tasks |
| 31 | 9331910 | Menon et al. (1997) | Combined event-related fMRI and EEG evidence for temporal-parietal cortex activation during target detection | Yes |  |
| 32 | 31400532 | Moore et al. (2019) | Integration of spatio-temporal dynamics in emotion-cognition interactions: A simultaneous fMRI-ERP investigation using the emotional oddball task | No | Wrong contrast: Novel |
| 33 | 12575464 | Mulert et al. (2002) | Simultaneous ERP and event-related fMRI: focus on the time course of brain activity in target detection | No | No coordinates available |
| 34 | 15109999 | Mulert et al. (2004) | Integration of fMRI and simultaneous EEG: towards a comprehensive understanding of localization and time-course of brain activity in target detection | Yes |  |
| 35 | 14502083 | Müller et al. (2003) | Sparse imaging of the auditory oddball task with functional MRI | Yes |  |
| 36 | 15820638 | Otzenberger et al. (2005) | P300 recordings during event-related fMRI: a feasibility study | No | Wrong contrast: Target-novel |
| 37 | 17943000 | Petit et al. (2007) | Right hemisphere dominance for auditory attention and its modulation by eye position: an event related fMRI study | Yes |  |
| 38 | 22102362 | Sabri et al. (2013) | Neural events leading to and associated with detection of sounds under high processing load | Yes |  |
| 39 | 18838100 | Schofield et al. (2009) | Disturbances in selective information processing associated with the BDNF Val66Met polymorphism: evidence from cognition, the P300 and fronto-hippocampal systems | No | Methodology: Genetic groups contrast |
| 40 | 17136467 | Shahin et al. (2006) | Scalp topography and intracerebral sources for ERPs recorded during auditory target detection | No | No coordinates available |
| 41 | 10913710 | Stevens et al. (2000) | Event-related fMRI of auditory and visual oddball tasks | Yes |  |
| 42 | 16364059 | Stevens et al. (2005) | fMRI in an oddball task: effects of target-to-target interval | No | Wrong contrast: Target to target interval |
| 43 | 15955488 | Stevens et al. (2005) | Hemispheric differences in hemodynamics elicited by auditory oddball stimuli | No | Methodology: Hemispheric comparation |
| 44 | 10988036 | Strange et al. (2000) | Brain mechanisms for detecting perceptual, semantic, and emotional deviance | No | Wrong analysis: Conjuction |
| 45 | 11595101 | Vouloumanos et al. (2001) | Detection of sounds in the auditory stream: event-related fMRI evidence for differential activation to speech and nonspeech | No | Incomplete data, no answer from authors after contact |
| 46 | 25797833 | Walz et al. (2015) | Prestimulus EEG alpha oscillations modulate task-related fMRI BOLD responses to auditory stimuli | No | No coordinates available |
| 47 | 24305817 | Walz et al. (2013) | Simultaneous EEG-fMRI reveals temporal evolution of coupling between supramodal cortical attention networks and the brainstem | No | Methodology: EEG and BOLD |
| 48 | 19505583 | Warbrick et al. (2009) | Single-trial P3 amplitude and latency informed event-related fMRI models yield different BOLD response patterns to a target detection task | Yes |  |
| 49 | 22649231 | Wessel et al. (2012) | Surprise and error: common neuronal architecture for the processing of errors and novelty | No | Wrong contrast: novel-standard |
| 50 | 10501557 | Yoshiura et al. (1999) | Functional MRI study of auditory and visual oddball tasks | No | Incomplete data, no answer from authors after contact |

## Included studies

**Timing studies**

Coull JT, Vidal F, Nazarian B, Macar F. Functional anatomy of the attentional modulation of time estimation. *Science.* (2004) 303:1506-8. doi:10.1126/science.1091573.

Coull JT, Nazarian B, Vidal F. Timing, storage, and comparison of stimulus duration engage discrete anatomical components of a perceptual timing network. *J Cogn Neurosci.* (2008) 20:2185-97. doi:10.1162/jocn.2008.20153.

Coull JT, Hwang HJ, Leyton M, Dagher A. Dopamine precursor depletion impairs timing in healthy volunteers by attenuating activity in putamen and supplementary motor area. *J Neurosci.* (2012) 32:16704-15. doi:10.1523/JNEUROSCI.1258-12.2012.

Lewis PA, Miall RC. Brain activation patterns during measurement of sub- and supra-second intervals. *Neuropsychologia.* (2003) 41:1583-92. doi:10.1016/s0028-3932(03)00118-0.

Livesey AC, Wall MB, Smith AT. Time perception: manipulation of task difficulty dissociates clock functions from other cognitive demands. *Neuropsychologia* (2007) 45:321-doi: 10.1016/j.neuropsychologia.2006.06.033.

Morillon B, Kell CA, Giraud AL. Three stages and four neural systems in time estimation. *J Neurosci.* (2009) 29:14803-11. doi:10.1523/JNEUROSCI.3222-09.2009.

Pfeuty M, Dilharreguy B, Gerlier L, Allard M. fMRI identifies the right inferior frontal cortex as the brain region where time interval processing is altered by negative emotional arousal. *Hum Brain Mapp.* (2015) 36:981-95. doi:10.1002/hbm.22680.

Pouthas V, George N, Poline JB, Pfeuty M, Vandemoorteele PF, Hugueville L, Ferrandez AM, Lehéricy S, Lebihan D, Renault B. Neural network involved in time perception: an fMRI study comparing long and short interval estimation. *Hum Brain Mapp.* (2005) 25:433-41. doi: 10.1002/hbm.20126.

Rao SM, Mayer AR, Harrington DL. The evolution of brain activation during temporal processing. *Nat Neurosci.* (2001) 4(3):317-23. doi:10.1038/85191.

Smith A, Taylor E, Lidzba K, Rubia K. A right hemispheric frontocerebellar network for time discrimination of several hundreds of milliseconds. *Neuroimage* (2003) 20:344-350. doi: 10.1016/s1053-8119(03)00337-9.

Wiener M, Lee YS, Lohoff FW, Coslett HB. Individual differences in the morphometry and activation of time perception networks are influenced by dopamine genotype. *Neuroimage.* (2014) 89:10-22. doi: 10.1016/j.neuroimage.2013.11.019.

**Oddball studies**

1. Eichele T, Specht K, Moosmann M, Jongsma ML, Quiroga RQ, Nordby H, Hugdahl K. Assessing the spatiotemporal evolution of neuronal activation with single-trial event-related potentials and functional MRI. *Proc Natl Acad Sci U S A.* (2005) 102:17798-803. doi: 10.1073/pnas.0505508102.
2. Fajkus J, Mikl M, Shaw DJ, Brázdil M. An fMRI investigation into the effect of preceding stimuli during visual oddball tasks. *J. Neurosci. Methods* (2015) 251:56-61. doi: 10.1016/j.jneumeth.2015.05.005.
3. Friedman D, Goldman R, Stern Y, Brown TR. The brain's orienting response: An event-related functional magnetic resonance imaging investigation. *Hum Brain Mapp*. (2009) 30:1144-54. doi: 10.1002/hbm.20587.
4. Gur RC, Turetsky BI, Loughead J, Waxman J, Snyder W, Ragland JD, et al. Hemodynamic responses in neural circuitries for detection of visual target and novelty: an event-related fMRI study. *Hum. Brain Mapp*. (2007) 28:263-274. doi:10.1002/hbm.20319.

1. Horovitz SG, Skudlarski P, Gore JC. Correlations and dissociations between BOLD signal and P300 amplitude in an auditory oddball task: a parametric approach to combining fMRI and ERP. *Magn Reson Imaging.* (2002) 20:319-25. doi: 10.1016/s0730-725x(02)00496-4.
2. Huettel SA, Misiurek J, Jurkowski AJ, McCarthy G. Dynamic and strategic aspects of executive processing. *Brain Res.* (2004) 1000:78-84. doi: 10.1016/j.brainres.2003.11.041.
3. Linden DE, Prvulovic D, Formisano E, Völlinger M, Zanella FE, Goebel R, et al. The functional neuroanatomy of target detection: an fMRI study of visual and auditory oddball tasks. *Cereb. Cortex****.*** (1999) 9:815-823. doi:10.1093/cercor/9.8.815.
4. Mantini D, Corbetta M, Perrucci MG, Romani GL, del Gratta C. Large-scale brain networks account for sustained and transient activity during target detection. *Neuroimage* (2009) 44: 265-274. doi:10.1016/j.neuroimage.2008.08.019.
5. Menon V, Ford JM, Lim KO, Glover GH, Pfefferbaum A. Combined event-related fMRI and EEG evidence for temporal-parietal cortex activation during target detection. *Neuroreport.* (1997) 8:3029-37. doi:10.1097/00001756-199709290-00007.
6. Mulert C, Jäger L, Schmitt R, Bussfeld P, Pogarell O, Möller HJ, et al. Integration of fMRI and simultaneous EEG: towards a comprehensive understanding of localization and time-course of brain activity in target detection. *Neuroimage* (2004) 22:83-94. doi:10.1016/j.neuroimage.2003.10.051.
7. Müller BW, Stude P, Nebel K, Wiese H, Ladd ME, Forsting M, et al. Sparse imaging of the auditory oddball task with functional MRI. *Neuroreport.* (2003) 14:1597-601. doi: 10.1097/00001756-200308260-00011.
8. Petit L, Simon G, Joliot M, Andersson F, Bertin T, Zago L, et al. Right hemisphere dominance for auditory attention and its modulation by eye position: an event related fMRI study. *Restor. Neurol. Neurosci*. (2007) 25:211-225.
9. Sabri M, Humphries C, Binder JR, Liebenthal E. Neural events leading to and associated with detection of sounds under high processing load. *Hum Brain Mapp.* (2013) 34:587-97. doi: 10.1002/hbm.21457.
10. Stevens AA, Skudlarski P, Gatenby JC, Gore JC. Event-related fMRI of auditory and visual oddball tasks. *Magn. Reson. Imaging*. (2000) 18:495-502. doi:10.1016/s0730-725x(00)00128-4.
11. Warbrick T, Mobascher A, Brinkmeyer J, Musso F, Richter N, Stoecker T, Fink GR, Shah NJ, Winterer G. Single-trial P3 amplitude and latency informed event-related fMRI models yield different BOLD response patterns to a target detection task. *Neuroimage.* (2009) 47:1532-44. doi: 10.1016/j.neuroimage.2009.05.082.

# Supplementary Figures and Tables

## Timing studies SDM-PSI Meta-analysis

| MNI coordinate | | SDM-Z | P | Voxels | Description |
| --- | --- | --- | --- | --- | --- |
| **52,28,4** | 5.545 | 0.000999987 | 1487 | Right inferior frontal gyrus, triangular part, BA 45 |  |
|  | **Local peaks:** | | | |  |
|  | MNI coordinate | SDM-Z | P | Description |  |
|  | 52,28,4 | 5.545 | 0.000999987 | Right inferior frontal gyrus, triangular part, BA 45 |  |
|  | 48,26,6 | 5.037 | 0.000999987 | Right inferior frontal gyrus, triangular part, BA 45 |  |
|  | 60,16,8 | 2.965 | 0.003000021 | Right inferior frontal gyrus, opercular part, BA 44 |  |
|  | 32,16,10 | 2.849 | 0.012000024 | Right insula, BA 48 |  |
|  | 50,26,20 | 2.845 | 0.012000024 | Right inferior frontal gyrus, triangular part, BA 45 |  |
| MNI coordinate | | SDM-Z | P | Voxels | Description |
| **26,42,30** | 4.668 | 0.000999987 | 678 | Right middle frontal gyrus, BA 46 |  |
|  | **Local peaks:** | | | |  |
|  | MNI coordinate | SDM-Z | P | Description |  |
|  | 26,42,30 | 4.668 | 0.000999987 | Right middle frontal gyrus, BA 46 |  |
|  | 40,42,24 | 3.634 | 0.003000021 | Right middle frontal gyrus, BA 46 |  |
|  | 40,50,12 | 3.320 | 0.028999984 | Right middle frontal gyrus, BA 46 |  |
|  | 34,54,6 | 3.051 | 0.032000005 | Right middle frontal gyrus, BA 10 |  |
|  | 40,52,8 | 2.976 | 0.031000018 | Right middle frontal gyrus, BA 46 |  |
|  | 42,46,12 | 2.891 | 0.033999979 | Right middle frontal gyrus, BA 45 |  |
|  | 32,54,14 | 2.847 | 0.026000023 | Right middle frontal gyrus, BA 46 |  |
|  | 28,54,24 | 2.664 | 0.027999997 | Right middle frontal gyrus, BA 46 |  |
| Blobs of ≥ 678 voxels with all voxels SDM-Z ≥ 2.385 and all peaks SDM-Z ≥ 4.668 | | | | |  |

## Oddball studies SDM-PSI Meta-analysis

| MNI coordinate | SDM-Z | P | Voxels | Description |
| --- | --- | --- | --- | --- |
| **40,-8,12** | 6.095 | 0.000999987 | 19652 | Right insula, BA 48 |
|  | **Local peaks:** | | | |
|  | MNI coordinate | SDM-Z | P | Description |
|  | 40,-8,12 | 6.095 | 0.000999987 | Right insula, BA 48 |
|  | 54,-28,44 | 5.868 | 0.000999987 | Right supramarginal gyrus, BA 2 |
|  | 56,-14,40 | 5.616 | 0.000999987 | Right postcentral gyrus, BA 3 |
|  | 56,-18,42 | 5.577 | 0.000999987 | Right postcentral gyrus, BA 3 |
|  | 48,-4,8 | 5.505 | 0.000999987 | Right rolandic operculum, BA 48 |
|  | 44,-6,48 | 5.260 | 0.000999987 | Right precentral gyrus, BA 6 |
|  | 38,10,2 | 5.241 | 0.000999987 | Right insula, BA 48 |
|  | 42,-10,0 | 5.159 | 0.000999987 | Right insula, BA 48 |
|  | 48,0,8 | 5.103 | 0.000999987 | Right rolandic operculum, BA 48 |
|  | 50,-22,48 | 4.959 | 0.000999987 | Right postcentral gyrus, BA 3 |
|  | 54,-20,48 | 4.952 | 0.000999987 | Right postcentral gyrus, BA 4 |
|  | 46,-16,50 | 4.903 | 0.000999987 | Right precentral gyrus, BA 6 |
|  | 30,12,2 | 4.867 | 0.000999987 | Right lenticular nucleus, putamen, BA 48 |
|  | -4,-74,2 | 4.857 | 0.000999987 | Left lingual gyrus, BA 17 |
|  | 58,-20,12 | 4.731 | 0.000999987 | Right superior temporal gyrus, BA 48 |
|  | 14,-66,32 | 4.730 | 0.001999974 | Right precuneus |
|  | 48,4,14 | 4.653 | 0.000999987 | Right frontal aslant tract |
|  | 48,6,20 | 4.635 | 0.000999987 | Right inferior frontal gyrus, opercular part, BA 44 |
|  | 32,-10,58 | 4.615 | 0.000999987 | Right superior frontal gyrus, dorsolateral, BA 6 |
|  | 46,-12,58 | 4.499 | 0.000999987 | Right precentral gyrus, BA 4 |
|  | -6,-68,0 | 4.493 | 0.000999987 | Left lingual gyrus, BA 18 |
|  | -8,-72,10 | 4.445 | 0.000999987 | (undefined) |
|  | 62,-22,6 | 4.379 | 0.000999987 | Corpus callosum |
|  | 42,46,14 | 4.346 | 0.000999987 | Right middle frontal gyrus, BA 45 |
|  | 54,-22,16 | 4.300 | 0.000999987 | Right rolandic operculum, BA 48 |
|  | 40,50,14 | 4.295 | 0.000999987 | Right middle frontal gyrus, BA 46 |
|  | 42,46,10 | 4.270 | 0.000999987 | Right middle frontal gyrus, BA 45 |
|  | 60,-42,20 | 4.177 | 0.000999987 | Right superior temporal gyrus, BA 42 |
|  | 54,-50,48 | 4.091 | 0.000999987 | Right inferior parietal (excluding supramarginal and angular) gyri, BA 40 |
|  | 56,-4,-4 | 4.070 | 0.000999987 | Corpus callosum |
|  | 40,44,20 | 4.052 | 0.000999987 | Right middle frontal gyrus, BA 45 |
|  | 38,48,20 | 4.027 | 0.000999987 | Right middle frontal gyrus, BA 45 |
|  | 50,-48,48 | 4.009 | 0.000999987 | Right inferior parietal (excluding supramarginal and angular) gyri, BA 40 |
|  | 50,-44,46 | 3.922 | 0.000999987 | Right inferior parietal (excluding supramarginal and angular) gyri, BA 40 |
|  | 64,-6,24 | 3.920 | 0.000999987 | Right postcentral gyrus, BA 43 |
|  | 54,-30,6 | 3.829 | 0.000999987 | Corpus callosum |
|  | 58,-36,2 | 3.764 | 0.000999987 | Right middle temporal gyrus, BA 22 |
|  | 64,-30,2 | 3.762 | 0.000999987 | Right superior temporal gyrus, BA 21 |
|  | 40,16,-14 | 3.725 | 0.000999987 | Right insula, BA 38 |
|  | 56,-12,16 | 3.721 | 0.000999987 | Right rolandic operculum, BA 48 |
|  | 54,-36,20 | 3.718 | 0.000999987 | Right superior temporal gyrus, BA 42 |
|  | 62,-8,18 | 3.711 | 0.000999987 | Right postcentral gyrus, BA 43 |
|  | 44,22,-8 | 3.706 | 0.000999987 | Right insula, BA 47 |
|  | 62,-36,10 | 3.675 | 0.000999987 | Right superior temporal gyrus, BA 42 |
|  | 54,-40,22 | 3.674 | 0.000999987 | Right arcuate network, posterior segment |
|  | 64,-32,-2 | 3.669 | 0.000999987 | Right middle temporal gyrus, BA 21 |
|  | 62,-40,0 | 3.655 | 0.000999987 | Right middle temporal gyrus, BA 22 |
|  | 64,-34,2 | 3.654 | 0.000999987 | Right middle temporal gyrus, BA 21 |
|  | 62,-32,12 | 3.651 | 0.000999987 | Right superior temporal gyrus, BA 22 |
|  | 54,-36,24 | 3.612 | 0.000999987 | Right supramarginal gyrus, BA 48 |
|  | 52,-38,8 | 3.578 | 0.000999987 | Right superior temporal gyrus, BA 22 |
|  | 50,22,-2 | 3.557 | 0.000999987 | Right inferior frontal gyrus, triangular part, BA 47 |
|  | 2,-64,12 | 3.536 | 0.004000008 | Left calcarine fissure / surrounding cortex, BA 17 |
|  | 32,30,-2 | 3.526 | 0.000999987 | (undefined), BA 47 |
|  | 40,28,8 | 3.521 | 0.000999987 | Right superior longitudinal fasciculus III |
|  | 46,24,32 | 3.458 | 0.000999987 | Right middle frontal gyrus, BA 44 |
|  | 56,2,12 | 3.451 | 0.000999987 | Right rolandic operculum, BA 48 |
|  | 50,24,2 | 3.429 | 0.000999987 | Right inferior frontal gyrus, triangular part, BA 45 |
|  | 68,-28,12 | 3.421 | 0.000999987 | Right superior temporal gyrus, BA 22 |
|  | 42,14,-20 | 3.412 | 0.000999987 | Right temporal pole, superior temporal gyrus |
|  | 40,28,28 | 3.382 | 0.000999987 | Right inferior frontal gyrus, triangular part, BA 45 |
|  | 58,10,-10 | 3.374 | 0.000999987 | Right temporal pole, superior temporal gyrus, BA 38 |
|  | 46,28,0 | 3.368 | 0.000999987 | Right inferior frontal gyrus, triangular part, BA 47 |
|  | 44,28,-4 | 3.323 | 0.000999987 | Right inferior frontal gyrus, orbital part, BA 47 |
|  | 66,-24,20 | 3.273 | 0.000999987 | Right supramarginal gyrus, BA 22 |
|  | 32,26,8 | 3.246 | 0.000999987 | Right insula, BA 48 |
|  | 0,-66,-8 | 3.241 | 0.005999982 | Cerebellum, vermic lobule VI |
|  | 10,-68,50 | 3.193 | 0.009999990 | Right precuneus, BA 7 |
|  | 52,-2,18 | 3.192 | 0.000999987 | Right arcuate network, anterior segment |
|  | 8,-66,44 | 3.191 | 0.009999990 | Right precuneus, BA 7 |
|  | 48,36,30 | 3.151 | 0.000999987 | Right middle frontal gyrus, BA 45 |
|  | 6,-74,38 | 3.132 | 0.009999990 | Right precuneus, BA 7 |
|  | 4,-68,-10 | 3.105 | 0.008000016 | Cerebellum, vermic lobule VI, BA 18 |
|  | 48,18,26 | 3.097 | 0.000999987 | Right inferior frontal gyrus, opercular part, BA 48 |
|  | 42,28,0 | 3.053 | 0.000999987 | Right inferior frontal gyrus, triangular part, BA 47 |
|  | 8,-74,42 | 3.013 | 0.009999990 | Right precuneus, BA 7 |
|  | 38,36,12 | 3.012 | 0.000999987 | Right superior longitudinal fasciculus III |
|  | 48,6,38 | 2.965 | 0.000999987 | Right precentral gyrus, BA 6 |
|  | 12,-80,46 | 2.938 | 0.015999973 | Right precuneus, BA 7 |
|  | 12,-82,36 | 2.918 | 0.015999973 | Right cuneus cortex, BA 19 |
|  | 48,-6,-16 | 2.884 | 0.000999987 | Right middle temporal gyrus, BA 21 |
|  | -8,-84,14 | 2.875 | 0.010999978 | Corpus callosum |
|  | -2,-74,28 | 2.862 | 0.027000010 | Left cuneus cortex |
|  | 64,-24,-10 | 2.848 | 0.000999987 | Right middle temporal gyrus, BA 21 |
|  | -4,-74,24 | 2.795 | 0.027000010 | Left cuneus cortex |
|  | -4,-80,26 | 2.677 | 0.027000010 | Left cuneus cortex, BA 18 |
|  | 28,-60,36 | 2.616 | 0.035000026 | Corpus callosum |
|  | -2,-80,30 | 2.568 | 0.035000026 | Left cuneus cortex, BA 18 |
|  | -8,-72,40 | 2.560 | 0.035000026 | Left precuneus, BA 7 |
|  | 26,-60,40 | 2.519 | 0.035000026 | Corpus callosum |
|  | 52,-64,32 | 2.490 | 0.004000008 | Right angular gyrus, BA 39 |
|  | 50,-58,32 | 2.477 | 0.004000008 | Right angular gyrus, BA 39 |
|  | 38,12,26 | 2.436 | 0.027000010 | Right inferior frontal gyrus, opercular part, BA 48 |
|  | 52,18,20 | 2.434 | 0.008000016 | Right inferior frontal gyrus, triangular part, BA 48 |
|  | 38,-56,48 | 2.375 | 0.015999973 | Right angular gyrus, BA 40 |
|  | 36,-74,40 | 2.349 | 0.035000026 | Right angular gyrus, BA 7 |
|  | 38,-62,48 | 2.295 | 0.027000010 | Right angular gyrus, BA 7 |
|  | 32,-66,54 | 2.283 | 0.049000025 | Right superior parietal gyrus, BA 7 |
|  | 34,-66,48 | 2.274 | 0.027000010 | Right angular gyrus, BA 7 |
|  | 26,-60,54 | 2.266 | 0.035000026 | Right superior parietal gyrus, BA 7 |
|  | 24,-72,42 | 2.130 | 0.050000012 | Right superior occipital gyrus, BA 7 |
|  | 26,-72,46 | 2.122 | 0.050000012 | Right superior occipital gyrus, BA 7 |
|  | 22,-70,46 | 2.061 | 0.050000012 | Right superior occipital gyrus, BA 7 |
| MNI coordinate | SDM-Z | P | Voxels | Description |
| **-34,-8,10** | 8.248 | ~0 | 13842 | Left insula, BA 48 |
|  | **Local peaks:** | | | |
|  | MNI coordinate | SDM-Z | P | Description |
|  | -34,-8,10 | 8.248 | ~0 | Left insula, BA 48 |
|  | -42,-32,48 | 7.796 | 0.000999987 | Left postcentral gyrus, BA 3 |
|  | -48,-30,46 | 7.742 | 0.000999987 | Left inferior parietal (excluding supramarginal and angular) gyri, BA 2 |
|  | -32,-12,8 | 7.728 | 0.000999987 | (undefined), BA 48 |
|  | -42,-28,46 | 7.588 | 0.000999987 | Left postcentral gyrus, BA 3 |
|  | -48,-24,46 | 7.552 | 0.000999987 | Left postcentral gyrus, BA 3 |
|  | -34,-10,2 | 7.325 | 0.000999987 | (undefined), BA 48 |
|  | -38,-12,-2 | 6.519 | 0.000999987 | Left insula, BA 48 |
|  | -58,-4,8 | 6.396 | 0.000999987 | Left rolandic operculum, BA 48 |
|  | -54,-18,34 | 6.090 | 0.000999987 | Left postcentral gyrus, BA 3 |
|  | -58,4,12 | 5.844 | 0.000999987 | Left inferior frontal gyrus, opercular part |
|  | -38,-14,52 | 5.831 | 0.000999987 | Left precentral gyrus, BA 6 |
|  | -56,8,12 | 5.805 | 0.000999987 | Left inferior frontal gyrus, opercular part, BA 6 |
|  | -54,12,10 | 5.796 | 0.000999987 | Left inferior frontal gyrus, opercular part, BA 48 |
|  | -38,-10,54 | 5.761 | 0.000999987 | Left precentral gyrus, BA 6 |
|  | -52,-6,12 | 5.755 | 0.000999987 | Left rolandic operculum, BA 48 |
|  | -46,-12,54 | 5.601 | 0.000999987 | Left postcentral gyrus, BA 6 |
|  | -58,-18,34 | 5.292 | 0.000999987 | Left postcentral gyrus |
|  | -56,0,18 | 5.291 | 0.000999987 | Left postcentral gyrus, BA 48 |
|  | -44,6,10 | 5.064 | 0.000999987 | Left frontal aslant tract |
|  | -46,2,12 | 5.058 | 0.000999987 | Left superior longitudinal fasciculus III |
|  | -42,10,2 | 4.954 | 0.000999987 | Left insula |
|  | -46,14,2 | 4.862 | 0.000999987 | Left inferior frontal gyrus, opercular part, BA 48 |
|  | -58,-10,30 | 4.856 | 0.000999987 | Left postcentral gyrus, BA 43 |
|  | -54,0,34 | 4.792 | 0.000999987 | Left precentral gyrus, BA 6 |
|  | -52,-16,0 | 4.619 | 0.000999987 | Corpus callosum |
|  | -58,0,28 | 4.573 | 0.000999987 | Left precentral gyrus, BA 4 |
|  | -32,-52,52 | 4.449 | 0.000999987 | Left inferior parietal (excluding supramarginal and angular) gyri, BA 7 |
|  | -52,-12,30 | 4.387 | 0.000999987 | Left hand inferior U tract |
|  | -30,-18,64 | 4.076 | 0.000999987 | Left precentral gyrus, BA 6 |
|  | -28,-58,48 | 3.856 | 0.000999987 | Left inferior parietal (excluding supramarginal and angular) gyri, BA 7 |
|  | -60,-42,14 | 3.816 | 0.000999987 | Left superior temporal gyrus, BA 42 |
|  | -48,-48,46 | 3.791 | 0.000999987 | Left inferior parietal (excluding supramarginal and angular) gyri, BA 40 |
|  | -56,-32,12 | 3.787 | 0.000999987 | Left superior temporal gyrus, BA 42 |
|  | -46,-52,44 | 3.734 | 0.000999987 | Left inferior parietal (excluding supramarginal and angular) gyri, BA 40 |
|  | -60,-32,14 | 3.702 | 0.000999987 | Left superior temporal gyrus, BA 42 |
|  | -50,-46,28 | 3.510 | 0.000999987 | Left arcuate network, posterior segment |
|  | -50,-34,12 | 3.412 | 0.000999987 | Left superior temporal gyrus, BA 41 |
|  | -60,-28,20 | 3.408 | 0.000999987 | Left supramarginal gyrus, BA 48 |
|  | -48,-36,16 | 3.306 | 0.000999987 | Left superior temporal gyrus, BA 41 |
|  | -64,-32,18 | 3.279 | 0.000999987 | Left superior temporal gyrus, BA 42 |
|  | -36,16,-16 | 3.215 | 0.000999987 | Left inferior frontal gyrus, orbital part, BA 38 |
|  | -42,12,-16 | 3.080 | 0.000999987 | (undefined) |
|  | -56,-48,16 | 2.961 | 0.000999987 | Left superior temporal gyrus, BA 22 |
|  | -44,-26,20 | 2.907 | 0.000999987 | Left rolandic operculum, BA 48 |
|  | -62,-48,24 | 2.865 | 0.008000016 | Left supramarginal gyrus, BA 42 |
|  | -24,-42,58 | 2.695 | 0.000999987 | Corpus callosum |
|  | -52,-52,32 | 2.621 | 0.004000008 | Left angular gyrus, BA 40 |
|  | -60,-44,22 | 2.591 | 0.008000016 | Left superior temporal gyrus, BA 42 |
|  | -54,-52,26 | 2.546 | 0.004999995 | Left supramarginal gyrus, BA 22 |
|  | -52,-56,30 | 2.543 | 0.004999995 | Left angular gyrus, BA 39 |
|  | -34,-30,68 | 2.513 | 0.015999973 | Left postcentral gyrus, BA 4 |
|  | -60,-44,38 | 2.498 | 0.015999973 | Left inferior parietal (excluding supramarginal and angular) gyri, BA 40 |
|  | -62,-46,28 | 2.382 | 0.027000010 | Left supramarginal gyrus, BA 48 |
| MNI coordinate | SDM-Z | P | Voxels | Description |
| **12,4,42** | 6.779 | 0.000999987 | 5347 | Right median cingulate / paracingulate gyri |
|  | **Local peaks:** | | | |
|  | MNI coordinate | SDM-Z | P | Description |
|  | 12,4,42 | 6.779 | 0.000999987 | Right median cingulate / paracingulate gyri |
|  | 6,2,54 | 6.310 | 0.000999987 | Right supplementary motor area, BA 6 |
|  | 10,6,46 | 6.290 | 0.000999987 | Right supplementary motor area, BA 6 |
|  | 0,8,56 | 6.224 | 0.000999987 | Left supplementary motor area |
|  | 4,-6,32 | 5.944 | 0.000999987 | Right median cingulate / paracingulate gyri, BA 23 |
|  | 0,6,52 | 5.937 | 0.000999987 | Left supplementary motor area |
|  | 8,4,36 | 5.904 | 0.000999987 | Right median network, cingulum |
|  | -4,10,56 | 5.894 | 0.000999987 | Left supplementary motor area, BA 6 |
|  | 4,6,44 | 5.830 | 0.000999987 | Right median cingulate / paracingulate gyri, BA 24 |
|  | -2,0,58 | 5.809 | 0.000999987 | Left supplementary motor area |
|  | 0,10,40 | 5.781 | 0.000999987 | Left median cingulate / paracingulate gyri, BA 24 |
|  | 6,10,34 | 5.778 | 0.000999987 | Right median network, cingulum |
|  | 0,10,36 | 5.755 | 0.000999987 | Left median cingulate / paracingulate gyri, BA 24 |
|  | -2,-10,32 | 5.366 | 0.000999987 | Left median cingulate / paracingulate gyri, BA 23 |
|  | -8,2,38 | 5.318 | 0.000999987 | Left median cingulate / paracingulate gyri, BA 24 |
|  | -8,-4,40 | 5.184 | 0.000999987 | Left median cingulate / paracingulate gyri, BA 24 |
|  | -4,-10,40 | 5.176 | 0.000999987 | Left median cingulate / paracingulate gyri, BA 23 |
|  | -8,4,42 | 5.117 | 0.000999987 | Left median cingulate / paracingulate gyri, BA 24 |
|  | 4,-16,38 | 4.979 | 0.000999987 | Right median cingulate / paracingulate gyri, BA 23 |
|  | -6,-10,44 | 4.811 | 0.000999987 | Left median cingulate / paracingulate gyri |
|  | 4,-14,44 | 4.791 | 0.000999987 | Right median cingulate / paracingulate gyri, BA 23 |
|  | -4,-12,48 | 4.740 | 0.000999987 | Left supplementary motor area |
|  | -2,-26,28 | 3.747 | 0.000999987 | (undefined), BA 23 |
|  | -8,-28,36 | 3.478 | 0.000999987 | Left median network, cingulum |
|  | 2,-30,42 | 3.315 | 0.001999974 | Right median cingulate / paracingulate gyri, BA 23 |
|  | -2,-38,34 | 3.314 | 0.001999974 | Left median cingulate / paracingulate gyri, BA 23 |
|  | 10,-34,36 | 3.107 | 0.041000009 | Right median network, cingulum |
|  | 8,-38,38 | 2.790 | 0.041000009 | Right median cingulate / paracingulate gyri |
| MNI coordinate | SDM-Z | P | Voxels | Description |
| **-10,4,12** | 4.619 | 0.007000029 | 1193 | Left anterior thalamic projections |
|  | **Local peaks:** | | | |
|  | MNI coordinate | SDM-Z | P | Description |
|  | -10,4,12 | 4.619 | 0.007000029 | Left anterior thalamic projections |
|  | 2,-12,12 | 4.502 | 0.008000016 | (undefined) |
|  | -10,0,10 | 4.315 | 0.008000016 | Left anterior thalamic projections |
|  | -10,-4,8 | 4.211 | 0.008000016 | (undefined) |
|  | 4,-28,-4 | 4.203 | 0.009999990 | (undefined) |
|  | -12,4,18 | 4.180 | 0.009999990 | Left caudate nucleus |
|  | 2,-34,-4 | 4.105 | 0.009999990 | (undefined) |
|  | -2,-34,-4 | 4.011 | 0.010999978 | (undefined) |
|  | -2,-28,-6 | 3.897 | 0.015999973 | (undefined) |
|  | 10,-16,10 | 3.887 | 0.012000024 | Right thalamus |
|  | 6,-18,16 | 3.644 | 0.017000020 | Right anterior thalamic projections |
|  | 4,-18,4 | 3.362 | 0.023999989 | Right thalamus |
|  | -8,-28,-4 | 3.324 | 0.035000026 | (undefined) |
|  | -4,-8,4 | 3.278 | 0.023999989 | Left thalamus |
|  | -8,-24,2 | 3.180 | 0.041999996 | Left thalamus |
|  | -6,-20,14 | 3.004 | 0.038999975 | Left thalamus |
|  | -8,-26,10 | 2.968 | 0.038999975 | Left thalamus |
|  | -6,-20,-2 | 2.943 | 0.046000004 | Left anterior thalamic projections |
|  | 12,-2,12 | 2.904 | 0.042999983 | (undefined) |
|  | 12,0,16 | 2.861 | 0.044000030 | Right caudate nucleus |
| Blobs of ≥ 1193 voxels with all voxels SDM-Z ≥ 1.979 and all peaks SDM-Z ≥ 4.619 | | | | |

**2.3 Timing-Oddball SDM-PSI Multimodal**

| MNI coordinate | SDM-Z | P | Voxels | Description |
| --- | --- | --- | --- | --- |
| **48,16,4** | 3.740 | 0.000999987 | 1012 | Right inferior frontal gyrus, opercular part, BA 48 |
|  | **Local peaks:** | | |  |
|  | MNI coordinate | SDM-Z | P | Description |
|  | 48,16,4 | 3.740 | 0.000999987 | Right inferior frontal gyrus, opercular part, BA 48 |
|  | 44,18,0 | 3.632 | 0.000999987 | Right insula, BA 47 |
|  | 42,14,8 | 3.577 | 0.000999987 | Right fronto-insular tract 3 |
|  | 50,22,-2 | 3.557 | 0.000999987 | Right inferior frontal gyrus, triangular part, BA 47 |
|  | 50,24,2 | 3.429 | 0.000999987 | Right inferior frontal gyrus, triangular part, BA 45 |
|  | 46,28,0 | 3.368 | 0.000999987 | Right inferior frontal gyrus, triangular part, BA 47 |
|  | 40,28,6 | 3.302 | 0.000999987 | Right inferior frontal gyrus, triangular part, BA 47 |
|  | 46,26,-6 | 3.235 | 0.000999987 | Right inferior frontal gyrus, orbital part, BA 47 |
|  | 32,16,10 | 2.849 | 0.012000024 | Right insula, BA 48 |
|  | 52,18,18 | 2.263 | 0.015999973 | Right inferior frontal gyrus, triangular part, BA 48 |
| MNI coordinate | SDM-Z | P | Voxels | Description |
| **40,42,22** | 3.567 | 0.003000021 | 271 | Right middle frontal gyrus, BA 46 |
|  | **Local peaks:** | |  |  |
|  | MNI coordinate | SDM-Z | P | Description |
|  | 0,42,22 | 3.567 | 0.003000021 | Right middle frontal gyrus, BA 46 |
|  | 40,50,12 | 3.320 | 0.028999984 | Right middle frontal gyrus, BA 46 |
|  | 42,46,12 | 2.891 | 0.033999979 | Right middle frontal gyrus, BA 45 |
|  | 34,52,16 | 2.842 | 0.026000023 | Right middle frontal gyrus, BA 46 |
| Blobs of ≥ 271 voxels with all voxels SDM-Z ≥ 1.980 and all peaks SDM-Z ≥ 3.567 | | | | |

**
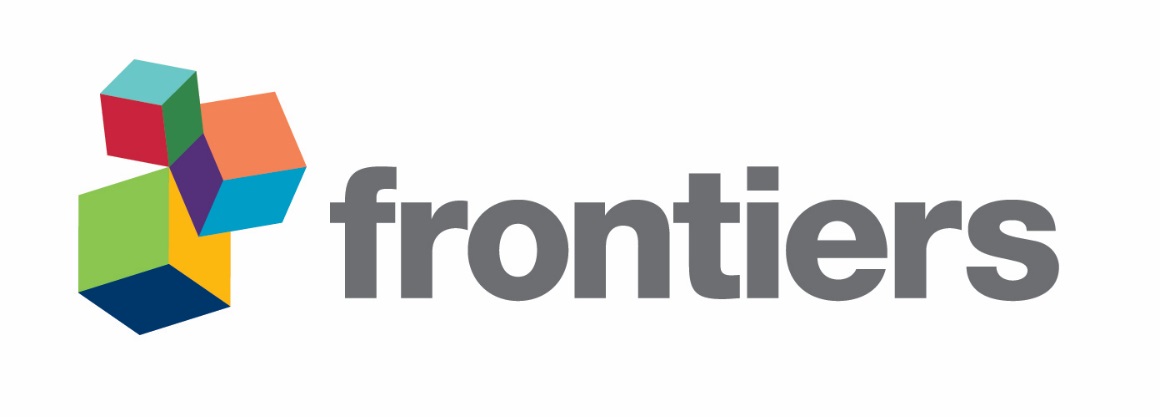
**
